# Circulating Selenium and Prostate Cancer Risk: A Mendelian Randomization Analysis

**DOI:** 10.1093/jnci/djy081

**Published:** 2018-05-17

**Authors:** James Yarmolinsky, Carolina Bonilla, Philip C Haycock, Ryan J Q Langdon, Luca A Lotta, Claudia Langenberg, Caroline L Relton, Sarah J Lewis, David M Evans, PRACTICAL Consortium, George Davey Smith, Richard M Martin

**Affiliations:** 1 MRC Integrative Epidemiology Unit, University of Bristol, Bristol, UK; 2 Population Health Sciences, Bristol Medical School, University of Bristol, Bristol, UK; 3 University Hospitals Bristol NHS Foundation Trust National Institute for Health Research Bristol Nutrition Biomedical Research Unit, University of Bristol, Bristol, UK; 4 MRC Epidemiology Unit, University of Cambridge, Cambridge, UK; 5 University of Queensland Diamantina Institute, Translational Research Institute, Brisbane, Australia

## Abstract

In the Selenium and Vitamin E Cancer Prevention Trial (SELECT), selenium supplementation (causing a median 114 μg/L increase in circulating selenium) did not lower overall prostate cancer risk, but increased risk of high-grade prostate cancer and type 2 diabetes. Mendelian randomization analysis uses genetic variants to proxy modifiable risk factors and can strengthen causal inference in observational studies. We constructed a genetic instrument comprising 11 single nucleotide polymorphisms robustly (*P* < 5 × 10^-8^) associated with circulating selenium in genome-wide association studies. In a Mendelian randomization analysis of 72 729 men in the PRACTICAL Consortium (44 825 case subjects, 27 904 control subjects), 114 μg/L higher genetically elevated circulating selenium was not associated with prostate cancer (odds ratio [OR] = 1.01, 95% confidence interval [CI] = 0.89 to 1.13). In concordance with findings from SELECT, selenium was weakly associated with advanced (including high-grade) prostate cancer (OR = 1.21, 95% CI = 0.98 to 1.49) and type 2 diabetes (OR = 1.18, 95% CI = 0.97 to 1.43; in a type 2 diabetes genome-wide association study meta-analysis with up to 49 266 case subjects and 249 906 control subjects). Our Mendelian randomization analyses do not support a role for selenium supplementation in prostate cancer prevention and suggest that supplementation could have adverse effects on risks of advanced prostate cancer and type 2 diabetes.

The development of interventions to prevent cancer requires robust causal knowledge, but few observational epidemiological claims are replicated in randomized controlled trials and some trial results are in the opposite direction to those seen observationally (ie, causing harm) (1,2). Such failures to translate observational findings into effective cancer prevention interventions arise in part because the limitations of observational research—confounding, reverse causation, and measurement error—preclude confident causal inference.

Mendelian randomization (MR) uses genetic variants as instruments (“proxies”) to assess whether a potential intervention target has a causal effect on a disease outcome in a nonexperimental (observational) setting (3). The use of genetic variants as proxies for intervention targets exploits the fact that germline genotype is largely independent of potential environmental or lifestyle factors. Further, because germline genetic variants are fixed at conception, MR analyses are not subject to reverse causation (Figure 1). An additional advantage of MR is that implementation does not require access to individual-level data or trait measurements in all samples: it can be implemented using summary information on gene exposure and gene outcome associations obtained from separate samples, which greatly increases the scope and efficiency of the approach (“two-sample Mendelian randomization”) (4,5).

**Figure 1.**
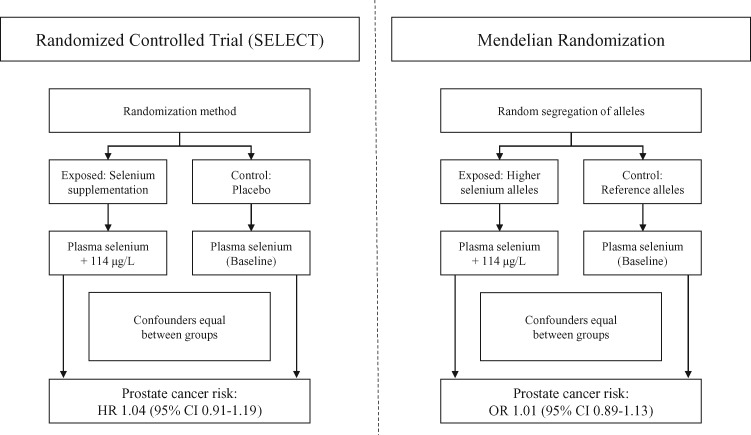
Schematic comparison of a randomized controlled trial (RCT; Selenium and Vitamin E Cancer Prevention Trial [SELECT]) to a Mendelian randomization analysis. In an RCT, individuals are randomly allocated to an intervention or control group (In SELECT, 200 μg/d selenium [114 μg/L increase in blood selenium] or placebo). If the trial is adequately sized, random assignment should ensure that intervention and control groups are comparable in all respects (eg, approximately equal distribution of potential confounding factors) except for the intervention being tested. In an intention-to-treat analysis, any observed differences in outcomes between intervention and control groups can then be attributed to the trial arm to which they were allocated. In a Mendelian randomization (MR) analysis, alleles that influence levels of a trait of interest are randomly allocated at conception. (In MR, the additive effects of selenium-raising alleles on 11 single nucleotide polymorphisms were scaled to mirror a 114 μg/L increase in blood selenium.) Groups defined by genotype should be comparable in all respects (eg, distribution of both genetic and environmental confounding factors) except for their exposure to a trait of interest. Any observed differences in outcomes between groups defined by genotype can then be attributed to differences in lifelong exposure to the trait of interest under study. Mendelian randomization is an application of the technique of instrumental variable (IV) analysis. In order for a genetic variant (or a multi-allelic instrument) to be used as an IV, three key assumptions must be met: 1) the instrument must be reliably associated with the exposure of interest, 2) the instrument should be independent of other factors affecting the outcome (confounders), and 3) the instrument should only affect the outcome through the exposure of interest (known as the exclusion restriction criterion). CI = confidence interval; HR = hazard ratio; SELECT = Selenium and Vitamin E Cancer Prevention Trial.

The largest ever prostate cancer prevention trial (SELECT, clinicaltrials.gov identifier: NCT00006392, n = 35 533) was designed to examine whether daily supplementation with selenium, vitamin E, or both agents combined could prevent prostate cancer (PCa) (6). It was abandoned at a cost of $114 million because of lack of efficacy compounded by possible carcinogenic (increased rates of high-grade [Gleason score ≥ 7] PCa) and adverse metabolic effects (increased rates of diabetes) of the interventions (6,7). We investigated whether Mendelian randomization could have predicted the results of the SELECT trial observed for selenium in a two-sample MR study of 72 729 individuals of European descent from the Prostate Cancer Association Group to Investigate Cancer Associated Alterations in the Genome (PRACTICAL) Consortium (8).

We obtained summary genome-wide association study (GWAS) statistics from analyses on 44 825 PCa case subjects and 27 904 control subjects from 108 studies in PRACTICAL. Summary statistics were also obtained from analyses on 6263 advanced PCa case subjects (Gleason ≥ 8, prostate-specific antigen > 100 ng/mL, metastatic disease [M1], or death from prostate cancer) and 27 235 control subjects. Written informed consent was obtained from all participants, and all studies in PRACTICAL have the relevant Institutional Review Board approval from each country, in accordance with the Declaration of Helsinki. Genotyping of PRACTICAL samples was performed using the Infinium OncoArray-500K, the details of which have been described previously (9).

To analyze the effect of selenium on type 2 diabetes (T2D), we used summary GWAS statistics from analyses in up to 49 266 T2D case subjects and 249 906 control subjects of European descent from a meta-analysis of the DIAbetes Genetics Replication And Meta-analysis (10), EPIC-InterAct (11), and UK Biobank (12). Methods for this meta-analysis have been published previously (13).

A genetic instrument to proxy for selenium was constructed by identifying single nucleotide polymorphisms (SNPs) robustly (*P* < 5×10^-8^) associated with selenium concentrations in a meta-analysis of blood and toenail selenium GWAS (14,15). Of 12 SNPs identified, one (rs558133) was not available in PRACTICAL, and thus 11 SNPs were used for both PCa analyses. As sensitivity analyses, we also constructed a restricted instrument using only SNPs robustly (*P* < 5×10^-8^) associated with selenium in a GWAS of blood selenium that were replicated (*P* < .05) in subsequent independent studies (14–16). For these analyses, of the five selenium SNPs initially identified, one (rs6859667) was not available in PRACTICAL, and thus four SNPs were used as instruments for both PCa analyses. All SNPs utilized and their corresponding ENSEMBL-mapped genes (17) are presented in the Supplementary Methods (available online).

We generated estimates of the proportion of variance in circulating selenium explained by our primary and restricted genetic instruments (*R*^2^) and F-statistics to examine the strength of our instruments, as outlined in the Supplementary Methods (available online), using methods previously described (18). Given that some of our SNPs were in mild linkage disequilibrium (LD) with each other, the ranges represent approximations of the total variance explained for our multi-allelic instruments calculated by summing the variance explained from lead SNPs at independent loci (using two different LD thresholds: *R*^2^ ≤ .01 and *R*^2^ ≤ .05) and thus may represent an underestimate of the variance explained of our instruments. The effect of our instrument on PCa (overall and advanced) and T2D was examined using a maximum-likelihood approach that adjusts for moderate correlation between variants (19). To compare the odds ratios from MR with the hazard ratios from SELECT, we estimated the odds ratios per 114 μg/L increase in circulating selenium to match measured pre- vs postintervention blood selenium differences between supplementation and control arms in SELECT (6). All statistical tests were two-sided. All statistical analyses were performed using R3.0.2.

Our primary genetic instrument (12 SNPs) explained from 2.5% to 5.0% of the variance in circulating selenium, and our restricted genetic instrument (five SNPs) explained from 2.1% to 3.3% of this variance. The corresponding F-statistic ranges for these instruments were 11.8–24.2 and 23.9–37.8, respectively, suggesting that our instruments were unlikely to suffer from weak instrument bias.

In Mendelian randomization analyses, a 114 μg/L increase in blood selenium was not associated with overall PCa risk (odds ratio [OR] = 1.01, 95% confidence interval [CI] = 0.89 to 1.13, z-test *P* = .93) (Table 1). Elevated selenium was weakly associated with advanced PCa (OR = 1.21, 95% CI = 0.98 to 1.49, *P* = .07) and T2D (OR = 1.18, 95% CI = 0.97 to 1.43, *P* = .11). Odds ratios for overall PCa, advanced PCa, and T2D were robust to sensitivity analyses, employing a restricted instrument (OR = 0.95, 95% CI = 0.80 to 1.14; OR = 1.09, 95% CI = 0.81 to 1.47; and OR = 1.23, 95% CI = 0.99 to 1.53; respectively).

**Table 1.** Comparison of the effect of 114 ug/L selenium on overall prostate cancer, high-grade/advanced prostate cancer, and type 2 diabetes in SELECT and Mendelian randomization

Outcome	SELECT HR (95% CI)	Mendelian randomization OR (95% CI)
Overall prostate cancer	1.04 (0.91 to 1.19)	1.01 (0.89 to 1.13)
High-grade/advanced prostate cancer*	1.21 (0.97 to 1.52)	1.21 (0.98 to 1.49)
Type 2 diabetes	1.07 (0.87 to 1.18)	1.18 (0.97 to 1.43)

* “High-grade prostate cancer” pertains to SELECT results, and “advanced prostate cancer” pertains to Mendelian randomization results. CI = confidence interval; HR = hazard ratio; OR = odds ratio.

The strengths of our analysis include the use of a multi-allelic score for circulating selenium (allowing us to increase the proportion of variance explained in circulating selenium) and the use of summary genetic association data from several large GWAS meta-analyses, which allowed us to increase the statistical power and precision of our analyses (20). Limitations of our analysis include that we were only able to directly examine the effect of selenium on advanced PCa and not high-grade PCa per se (summary estimates from PRACTICAL were available only for a composite “advanced” disease classification), thus preventing direct comparison with SELECT results. Additionally, our selenium SNPs were also associated with betaine, a putative risk factor for T2D (21), which could introduce horizontal pleiotropy into our analyses and violate MR assumptions (see the Figure 1 legend). Though we suspect that the association of these SNPs with selenium and betaine reflects the effect of selenium on betaine in the methionine cycle (22,23), and consequently that selenium and betaine likely influence T2D through the same biological pathway, we cannot rule out the possibility that at least part of a putative effect of selenium SNPs on T2D is through an alternate biological pathway involving betaine.

We conclude that, in contrast to findings from some prospective studies (24–29), our Mendelian randomization analysis using publicly available GWAS data did not find evidence for a causal effect of selenium on overall prostate cancer. Consistent with SELECT, we found weak evidence of a positive effect of selenium on advanced prostate cancer and type 2 diabetes risk. The alignment of MR with SELECT estimates mirrors the concordance of MR findings with other large, phase III trials, including adverse effects of elevated low-density lipoprotein cholesterol on coronary heart disease (30,31) and statins on T2D (32,33), as well as null effects of secretory phospholipase A(2)-IIA with cardiovascular disease (34–36) and high-density lipoprotein cholesterol on myocardial infarction (37–40). Mendelian randomization may serve as an important time-efficient and inexpensive first step in predicting both the efficacy and possible adverse effects of an intervention prior to the design of a randomized controlled trial.

## Funding

This work was supported by a Cancer Research UK program grant (C18281/A19169), Cancer Research UK Research PhD studentships (C18281/A20988 to JY and RJQL), and a Cancer Research UK Population Research Postdoctoral Fellowship (C52724/A20138 to PCH). This work was also supported by a three-year grant to identify modifiable risk factors for prostate cancer by the World Cancer Research Fund International (grant reference number: 2015/1421) to SJL. The Medical Research Council Integrative Epidemiology Unit at the University of Bristol is supported by the Medical Research Council (MC_UU_12013/1, MC_UU_12013/2, and MC_UU_12013/3) and the University of Bristol.

## Notes

Affiliations of authors: MRC Integrative Epidemiology Unit (JY, CB, PCH, RJQL, CLR, SJL, DME, GDS, RMM), Population Health Sciences, Bristol Medical School (JY, CB, PCH, RJQL, CLR, SJL, DME, GDS, RMM), and University Hospitals Bristol NHS Foundation Trust National Institute for Health Research Bristol Nutrition Biomedical Research Unit, University of Bristol, Bristol, UK (RMM); MRC Epidemiology Unit, University of Cambridge, Cambridge, UK (LAL, CL); University of Queensland Diamantina Institute, Translational Research Institute, Brisbane, Australia (DME).

The funders had no role in the design of the study; the collection, analysis, or interpretation of the data; the writing of the manuscript; or the decision to submit the manuscript for publication.

We are grateful to the contribution of data from EPIC-InterAct Investigators. The InterAct Study is led by Professor Nick Wareham at the MRC Epidemiology Unit, Cambridge, UK; InterAct funding was provided by the EU FP6 program (grant number LSHM_CT_2006_037197). A complete list of the principal investigators from the PRACTICAL Consortium and further funding acknowledgements can be found in the Supplementary Materials (available online).

## Supplementary Material

Supplementary Data
